# Polycomb Repressive Complex 2 is essential for development and maintenance of a functional TEC compartment

**DOI:** 10.1038/s41598-018-32729-z

**Published:** 2018-09-25

**Authors:** Nandini Singarapu, Keyue Ma, Kaitlin A. G. Reeh, Jianjun Shen, Jessica N. Lancaster, Song Yi, Huafeng Xie, Stuart H. Orkin, Nancy R. Manley, Lauren I. R. Ehrlich, Ning Jiang, Ellen R. Richie

**Affiliations:** 1 Department of Epigenetics and Molecular Carcinogenesis, The University of Texas M.D. Anderson Cancer Center, Science Park, Smithville, Texas 78957 USA; 2 Department of Biomedical Engineering, Cockrell School of Engineering, The University of Texas at Austin, Austin, TX 78712 USA; 3 Department of Molecular Biosciences, Institute for Cellular and Molecular Biology, The University of Texas at Austin, Austin, TX 78712 USA; 4 Department of Oncology, Dell Medical School and Department of Biomedical Engineering, Cockrell School of Engineering, The University of Texas at Austin, Austin, TX 78712 USA; 5 Division of Hematology/Oncology, Boston Children’s Hospital, Boston, MA 02115 USA; 6 Department of Pediatric Oncology, Dana-Farber Cancer Institute, Boston, MA 02115 USA; 7 Harvard Stem Cell Institute, Harvard University, Cambridge, MA 02138 USA; 8 Howard Hughes Medical Institute, Boston, MA 02115 USA; 9 Department of Genetics, Paul D. Coverdell Center, 500 DW Brooks Drive, University of Georgia, Athens, GA 30602 USA; 10 Livestrong Cancer Institutes, Dell Medical School, The University of Texas at Austin, Austin, TX 78712 USA

## Abstract

Thymic epithelial cells (TEC) are essential for thymocyte differentiation and repertoire selection. Despite their indispensable role in generating functional T cells, the molecular mechanisms that orchestrate TEC development from endodermal progenitors in the third pharyngeal pouch (3^rd^ PP) are not fully understood. We recently reported that the T-box transcription factor TBX1 negatively regulates TEC development. Although initially expressed throughout the 3^rd^ PP, *Tbx1* becomes downregulated in thymus-fated progenitors and when ectopically expressed impairs TEC progenitor proliferation and differentiation. Here we show that ectopic *Tbx1* expression in thymus fated endoderm increases expression of Polycomb repressive complex 2 (PRC2) target genes in TEC. PRC2 is an epigenetic modifier that represses gene expression by catalyzing trimethylation of lysine 27 on histone H3. The increased expression of PRC2 target genes suggests that ectopic *Tbx1* interferes with PRC2 activity and implicates PRC2 as an important regulator of TEC development. To test this hypothesis, we used *Foxn1*^*Cre*^ to delete *Eed*, a PRC2 component required for complex stability and function in thymus fated 3^rd^ PP endoderm. Proliferation and differentiation of fetal and newborn TEC were disrupted in the conditional knockout (*Eed*^*CKO*^) mutants leading to severely dysplastic adult thymi. Consistent with PRC2-mediated transcriptional silencing, the majority of differentially expressed genes (DEG) were upregulated in *Eed*^*CKO*^ TEC. Moreover, a high frequency of *Eed*^*CKO*^ DEG overlapped with DEG in TEC that ectopically expressed *Tbx1*. These findings demonstrate that PRC2 plays a critical role in TEC development and suggest that *Tbx1* expression must be downregulated in thymus fated 3^rd^ PP endoderm to ensure optimal PRC2 function.

## Introduction

T cell development occurs in the thymus where a unique three-dimensional network of thymic epithelial cells (TEC) provides indispensable signals for thymocyte growth, differentiation and T cell receptor repertoire selection. In mice, the epithelial thymic rudiment originates from bilaterally paired third pharyngeal pouches (3^rd^ PPs). Endodermal cells in the ventral domain of each 3^rd^ PP are specified to a thymus fate, whereas cells in the dorsal region are specified to a parathyroid fate [reviewed in^1–3^]. The transcription factors FOXN1 and GCM2 identify thymus- and parathyroid-fated cells respectively, and are required for their differentiation, but do not specify organ fate^4–6^. Multiple transcription factors and signaling pathways have been shown to play a role in 3^rd^ PP patterning [reviewed in^1–3^]. However, a complete picture of the transcriptional circuitry mediating organ specification and TEC development is still emerging. In this regard, we recently reported that the T-box transcription factor TBX1 plays a key role in 3^rd^ PP patterning^7^. Although initially expressed throughout the 3^rd^ PP, *Tbx1* expression is downregulated in the thymus-fated domain by embryonic day 10.5 (E10.5)^8,9^. To assess the consequences of inappropriate *Tbx1* expression, we used *Foxn1*^*Cre*^ to activate expression of an inducible *R26*^*iTbx*^ allele in thymus-fated 3^rd^ PP progenitors^7^. Ectopic TBX1 suppressed *Foxn1* expression, reduced proliferation and arrested differentiation of TEC progenitors, supporting the notion that *Tbx1* expression antagonizes TEC development and thymus organogenesis.

The dynamic, yet precisely timed changes in gene expression that underlie developmental programming are intimately linked to epigenetic control of transcription. Cross talk between transcription factors and chromatin modifying enzymes coordinates gene expression patterns to ensure correctly timed developmental transitions during organogenesis. Methylation, acetylation, and other posttranslational modifications of histone tails alter chromatin structure, thereby regulating accessibility to transcription factors [reviewed in^10,11^]. Several families of multisubunit protein complexes add or delete covalent histone modifications that alter chromatin compaction and regulate transcriptional activation. For example, members of the Trithorax group proteins activate transcription via H3K4 methylase activity, whereas Polycomb group repressive complex 1 (PRC1) and PRC2 repress transcription by catalyzing H2AK119 monoubiquitylation and H3K27 trimethylation respectively^10^. Although PRC1 and PRC2-mediated transcriptional regulation has been extensively studied in numerous developmental contexts, the role of these epigenetic modifiers in thymus organogenesis has received minimal attention. However, a recent report demonstrated that the absence of Cbx4, a core component of PRC1, impaired TEC proliferation and maturation^12^. Cbx4 was found to interact with p63, a transcription factor that regulates TEC progenitor proliferation and maintenance^12–14^. A connection between PRC1 and p63 was also reported in development of the epidermis where Cbx4 acts downstream of p63 to maintain epidermal progenitors^15^. Interestingly, a recent study reported high levels of H3K27me3 at transcriptional start sites of tissue-restricted genes that are promiscuously expressed in medullary TEC (mTEC) as a result of *Aire* expression^16^. These and other reported interactions between transcription factors and epigenetic regulators highlight the importance of cross-talk between chromatin modifying complexes and transcription factors in regulating cellular differentiation and function [reviewed in^17^].

Here we show that PRC2 is required for TEC development and maintenance and that an inverse relationship exists between *Tbx1* expression and PRC2 activity in TEC. Initially, we found increased expression of PRC2 target genes in *Foxn1*^*Cre*^;*R26*^*iTbx*^ TEC. This observation suggested that inappropriate expression of *Tbx1* in TEC progenitors interferes with PRC2-mediated transcriptional repression, which in turn may contribute to an aberrant thymus phenotype. To test the hypothesis that PRC2 plays a critical role in TEC development, we used *Foxn1*^*Cre*^ to conditionally delete *Eed*, which encodes a core component of the PRC2 complex required for its stability and function^18^. TEC proliferation and differentiation were severely disrupted in late fetal and early postnatal thymi leading to a profound loss in thymus cellularity and function. RNA-seq analysis revealed that the majority of differentially expressed genes (DEG) in *Foxn1*^*Cre*^;*Eed*^*fl/fl*^ TEC were upregulated, consistent with the canonical gene silencing function of PRC2. Interestingly, comparative gene expression profiling revealed considerable overlap between the DEG identified in *Foxn1*^*Cre*^;*Eed*^*fl/fl*^ TEC and those identified in *Foxn1*^*Cre*^;*R26*^*iTbx*^ TEC. Moreover, most of the shared DEG were dysregulated in the same direction strengthening the notion that ectopic *Tbx1* expression impairs TEC progenitors in part by interfering with PRC2 activity. Finally, consistent with the well-established role of PRC2 in suppressing expression of inappropriate lineage genes^19^, we found that transcriptional programs characteristic of non-TEC lineages are upregulated in *Foxn1*^*Cre*^;*Eed*^*fl/fl*^ TEC. Collectively, these findings establish that the epigenetic regulator PRC2 plays an indispensable role in ensuring proper TEC differentiation and lineage commitment.

## Results

### Differentially expressed genes in *Foxn1*^*Cre*/+^*;R26*^*iTbx*/+^ TEC include PRC2 targets

**Figure 1:**
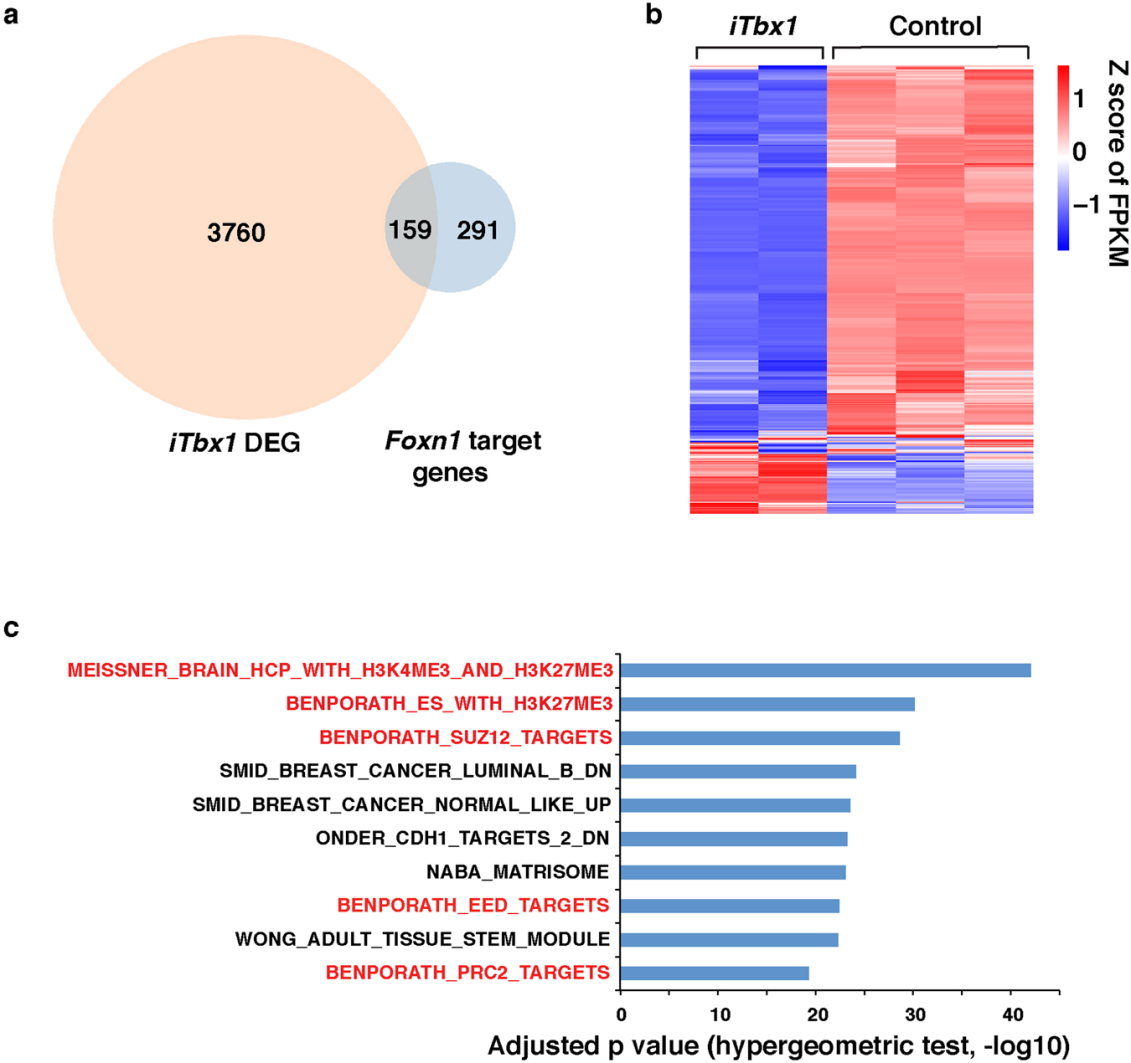
*iTbx1* DEG include *Foxn1* and PRC2 target genes. (**a**) Venn diagram showing overlap between *iTbx1* DEG and *Foxn1* target genes^21^. (**b**) Heatmap of *Foxn1* target genes in *iTbx1* and control TEC expressed as Z score of FPKM. (**c**) Bar graph showing enrichment for PRC2, EED, SUZ12 and H3K27me3 gene targets (red) in *iTbx1* mutant DEGs. The top 10 significantly enriched gene sets are plotted; the length of bar represents −log10 of the adjusted p value calculated using a hypergeometric test^57^.

Although *Tbx1* is initially expressed throughout the 3^rd^ PP endoderm, it is downregulated in the thymus-fated ventral domain by E10.5^5,9,20^. To better understand why *Tbx1* expression is extinguished in thymus fated 3^rd^ PP cells, we generated a conditional *R26*^*iTbx*/+^ allele and used *Foxn1*^*Cre*^ to activate ectopic *Tbx1* expression in thymus fated progenitors. As previously reported, TBX1 suppressed *Foxn1* expression in the ventral 3^rd^ PP and arrested TEC differentiation at an early progenitor stage^7^. In the current study, we investigated the molecular basis for this phenotype, by performing genome-wide transcriptome analysis on TEC isolated from E15.5 *Foxn1*^*Cre*/+^*;R26*^*iTbx*/+^ mutant (hereafter referred to as *iTbx1*) and control *Foxn1*^*Cre*/+^;*R26*^+/+^ thymi. RNA-seq analysis identified 3919 differentially expressed genes (DEG) based on a false discovery rate (FDR) <0.05 and fold change (FC) >2 (Fig. 1a and Table S1). DEG were equally partitioned between upregulated and downregulated genes. Consistent with our previous report showing that *Foxn1* expression is decreased in *iTbx1* TEC^7^, the RNA-seq data revealed that 159 of 450 high confidence *Foxn1* target genes^21^ were included in *iTbx1* DEG (Fig. 1a). Moreover, the majority of all *Foxn1* target genes were downregulated in *iTbx1* TEC (Fig. 1b and Table S2).

Unexpectedly, the RNA-seq analysis also showed that *iTbx1* DEG were highly enriched for target genes of PRC2, a multiprotein complex that deposits repressive H3K27me3 epigenetic marks^22,23^ (Fig. 1c). Furthermore, inspection of the ENCODE histone modification database showed that *iTbx1* DEG are enriched in genes that contain H3K27me3 modifications in various cell types (Fig. S1). The increased expression of PRC2 target genes in *iTbx1* TEC suggested that PRC2-mediated gene suppression may be compromised in *iTbx1* mutant TEC.

### Aberrant growth, maintenance and organization of *Eed*^*CKO*^ thymi is accompanied by reduced H3K27me3 marks in the TEC compartment

If the defects in *iTbx1* TEC differentiation and proliferation^7^ are due, at least in part, to diminished PRC2 activity, then inactivating PRC2 function might generate thymi with similar phenotypic characteristics. To test this prediction, we inactivated PRC2 activity by conditionally deleting *Eed* in TEC. EED is a scaffolding protein that stabilizes the PRC2 complex and is required for its catalytic activity^18,24,25^. We crossed *Eed*^*fl/fl*^^25^ and *Foxn1*^*Cre*^^26^ strains to generate *Foxn1*^*Cre*/+^;*Eed*^*fl/fl*^ progeny (hereafter referred to as *Eed*^*CKO*^) and Cre negative (*Foxn1*^+/+^;*Eed*^*fl/+*^ or *Foxn1*^+/+^;*Eed*^*fl/fl*^) controls. Efficient excision of *Eed* in *Eed*^*CKO*^ TEC was verified by PCR analysis (Fig. S2a). We validated the specificity of *Foxn1*^*Cre*^-mediated *Eed* deletion by flow cytometric analysis of a YFP reporter present in the *Eed*^*fl/fl*^ strain^25^. YFP was highly expressed in *Eed*^*CKO*^ TEC (defined as EpCAM+ CD45− cells), but was not detected in control TEC or *Eed*^*CKO*^ CD45+ thymocytes (Fig. S2b). Quantitative RT-PCR analysis showed that *Eed* mRNA expression was markedly reduced in *Eed*^*CKO*^ TEC by E14.5 and no longer detectable by E15.5 (Fig. S2c).

**Figure 2:**
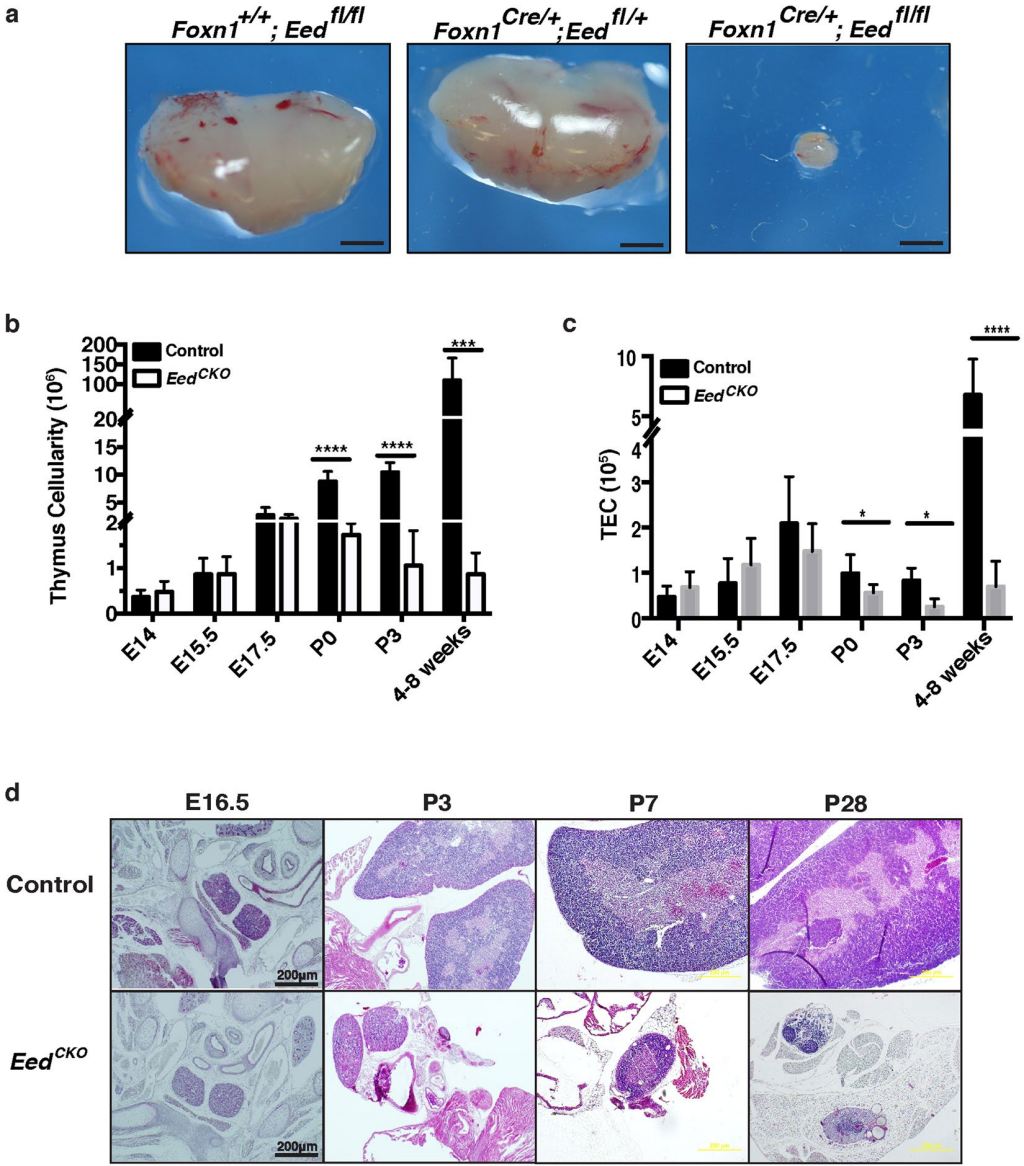
Deleting *Eed* in TEC results in progressively hypoplastic and disorganized thymi. (**a**) Gross appearance of thymi from 4 week old control (*Foxn1*^+/+^*Eed* ^*fl/fl*^), *Eed* haploinsufficient (*Foxn1*^*Cre/*+^*; Eed* ^*fl*/+^), and *Eed*^*CKO*^ (*Foxn1*^*Cre/*+^*; Eed* ^*fl/fl*^) mice. (**b**) Total thymus cellularity with age in control and *Eed*^*CKO*^ thymi. Each bar represents data from 3–13 individuals. Significance was calculated using an unpaired t test corrected for multiple comparisons using the Holm-Sidak method (*p < 0.05, **p < 0.01, ***p < 0.001, ****p < 0.0001). (**c**) TEC cellularity with age in control and *Eed*^*CKO*^ thymi. Significance is determined as in (**b**). (**d**) H&E stained sections from control and *Eed*^*CKO*^ mice at fetal (E16.5) and postnatal (P) day 3, 7 and 28. Images are at the same magnification; black bar shows scale (200 μm).

Homozygous deletion of *Eed* resulted in severely hypoplastic thymi, whereas *Eed* haploinsufficiency did not affect thymus size in 4 week old adult mice (Fig. 2a). To determine when thymus hypoplasia first manifests in *Eed*^*CKO*^ mice, we analyzed total and TEC cellularity in fetal, perinatal and adult *Eed*^*CKO*^ thymi (Fig. 2b). Despite the absence of detectable *Eed* mRNA by E15.5 (Fig. S2c), total thymus and TEC cellularity were not significantly reduced until the perinatal period (Fig. 2b,c). By the time *Eed*^*CKO*^ mice reached adulthood, total thymus and TEC cellularity were reduced by >90%. Similarly, thymus architecture was not obviously perturbed in *Eed*^*CKO*^ fetal thymic lobes (Fig. 2d). However, thymus architecture was severely disrupted in perinatal and adult *Eed*^*CKO*^ thymi. Histological analysis revealed perinatal *Eed*^*CKO*^ thymi to be highly cystic with poorly demarcated cortical and medullary regions. By 4 weeks of age, *Eed*^*CKO*^ thymi were profoundly hypoplastic and lacked any semblance of normal thymic architecture (Fig. 2d).

**Figure 3:**
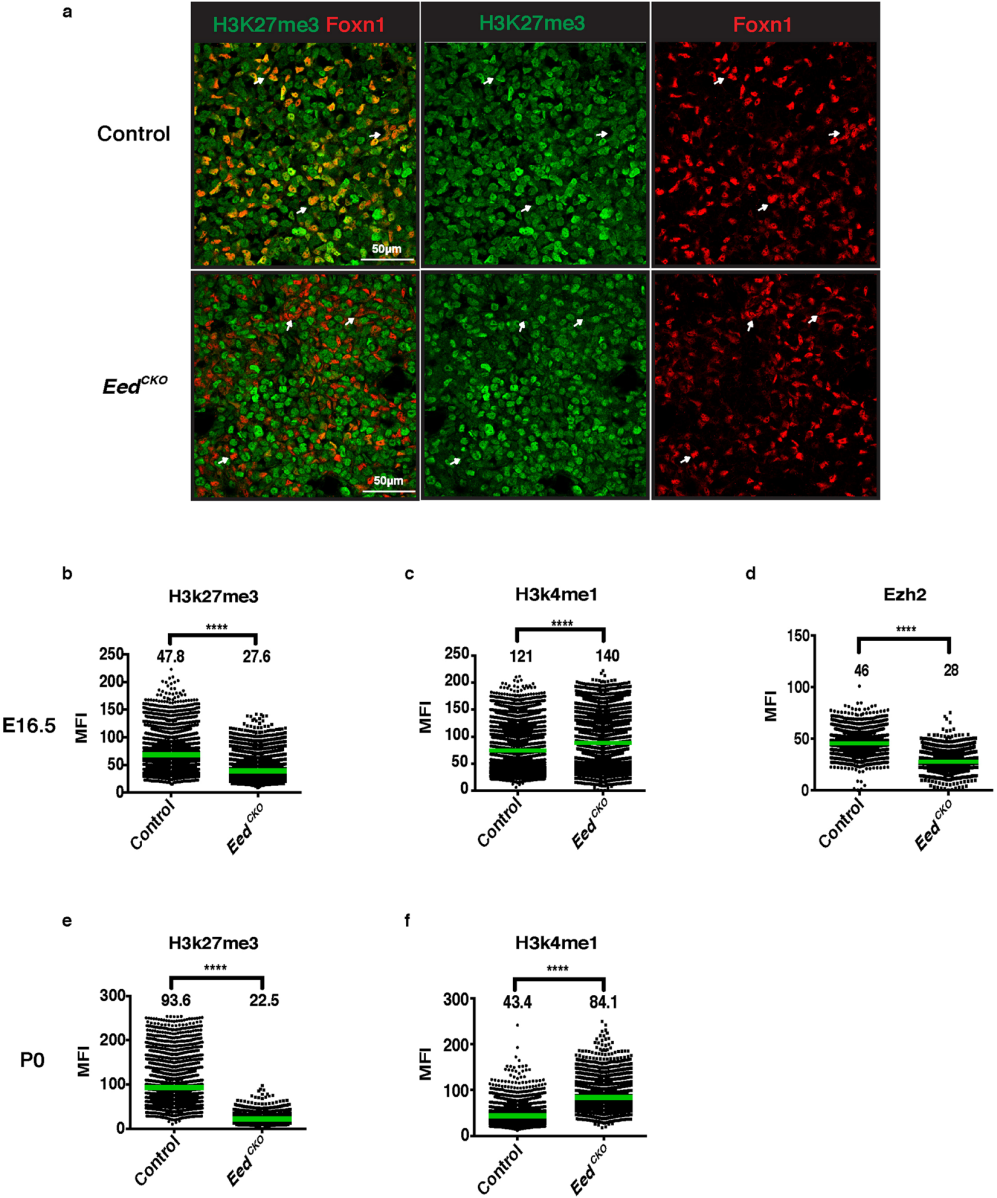
H3K27me3 levels are reduced in *Eed*^*CKO*^ TEC. (**a**) Representative sections from E16.5 thymic lobes co-stained for Foxn1 (red) and H3K27me3 (green). White arrows in the control sections show examples of Foxn1+ H3K27me3+ TEC. White arrows in the *Eed*^*CKO*^ sections show examples of Foxn1+ H3K27me3− TEC. (**b**,**e**) Quantitative analysis of MFI for H3K27me3 staining in E16.5 (**b**) and P0 (**e**) Foxn1+ control and *Eed*^*CKO*^ TEC. (**c**,**f**) Quantitative analysis of MFI for H3K4me1 staining in E16.5 (**c**) and P0 (**f**) Foxn1+ TEC. (**d**) Quantitative analysis of MFI for EZH2 staining in E16.5 Foxn1+ TEC.

The PRC2 complex suppresses gene expression by depositing repressive H3K27me3 histone marks. To evaluate H3K27me3 levels in fetal and newborn *Eed*^*CKO*^ and control TEC, we co-stained thymic sections with antibodies to H3K27me3 and FOXN1. Inspection of sections from E16.5 fetal thymic lobes revealed robust levels H3K27me3 staining in control Foxn1+ TEC, whereas H3K27me3 levels were clearly decreased in *Eed*^*CKO*^ TEC (Fig. 3a). Quantitative analysis of mean fluorescence intensity (MFI) confirmed lower levels of H3K27me3 in *Eed*^*CKO*^ compared to control E16.5 TEC and the reduction was even more severe in newborn (P0) TEC (Fig. 3b). In contrast, levels of the activating H3K4me1 mark were somewhat increased in control and *Eed*^*CKO*^ TEC, demonstrating a selective decrease in PRC2-regulated H3K27me3 levels (Fig. 3c). Consistent with previous reports showing that EED is required for PRC2 stability^18,24,25^, we also found that staining intensity for EZH2 was significantly reduced in *Eed*^*CKO*^ TEC (Fig. 3d).

### Eed deletion impairs TEC proliferation, survival and differentiation

**Figure 4:**
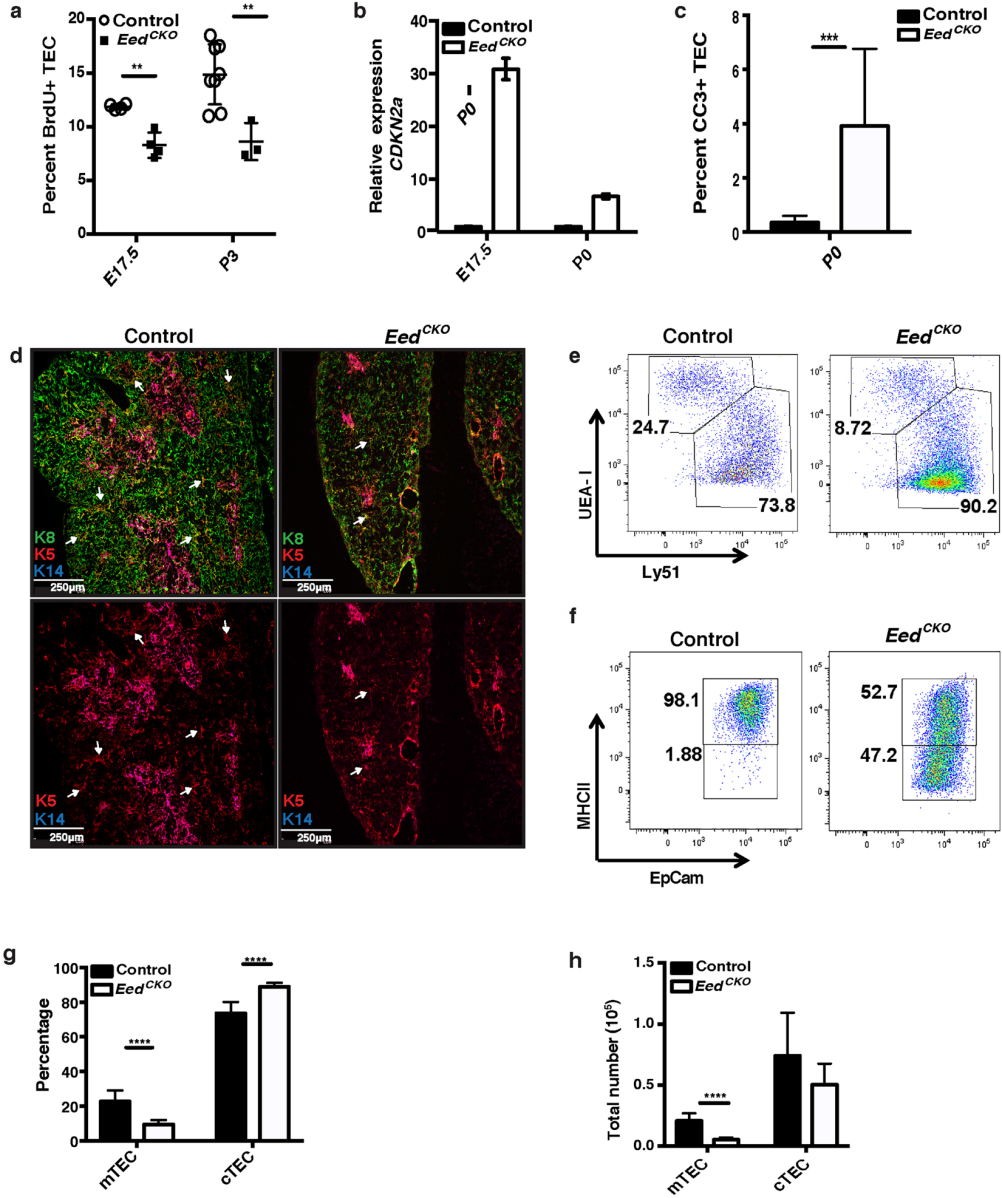
Eed deletion impairs TEC proliferation, survival and differentiation. (**a**) Percentage of fetal and newborn *Eed*^*CKO*^ versus control TEC that incorporate BrdU after injection into pregnant or newborn mice. (**b**) qRT-PCR analysis of *Cdkn2a* expression in E17.5 TEC isolated by FACS sorting. Expression in *Eed*^*CKO*^ TEC is normalized to control TEC. Each sample was analyzed in triplicate and the experiment was repeated twice. (**c**) Percentage of cleaved caspase 3 (CC3) positive Foxn1+ TEC in P0 *Eed*^*CKO*^ and control thymi. The frequency of apoptotic TEC was determined by counting the total number of Foxn1+ TEC that were positive for CC3 on each of 3 different sections/thymus. Results shown are from three independent experiments. (**d**) Frozen sections from P0 thymi were stained for K8 (green), K5 (red) and K14 (blue). White arrows indicate K8+K5+K14− TEC near the CMJ; this subset is widely distributed in controls, but sparse in mutant thymi. Note also the smaller medullary regions identified by K8−K5+K14+ TEC in *Eed*^*CKO*^ thymi. White bar indicates scale (250 μm). (**e**) Representative FACS plots of EpCAM+ CD45− TEC from P0 mice showing the distribution of UEA+Ly51− mTEC and UEA− Ly51+ cTEC subsets in control and *Eed*^*CKO*^ thymi. (**f**) Representative FACS plots showing expansion of the MHCII^lo^ TEC subset in P0 *Eed*^*CKO*^ thymi. (**g**) Bar graph showing percentage (mean ± SD) of cTEC and mTEC subsets in control and *Eed*^*CKO*^ thymi. (**h**) Bar graph showing number (mean ± SD) of cTEC and mTEC in control and *Eed*^*CKO*^ thymi. Significance was calculated using an unpaired t test corrected for multiple comparisons using the Holm-Sidak method (*p < 0.05, **p < 0.01, ***p < 0.001, ****p < 0.0001).

Additional analyses were performed to determine if the aberrant thymus phenotype in *Eed*^*CKO*^ mice is a consequence of defects in TEC proliferation, survival and/or differentiation. Previous reports have shown that the absence of functional PRC2 reduces proliferation of skin, intestinal and hematopoietic progenitor cells^25,27–30^. These studies linked decreased proliferation to increased expression of *Cdkn2a*, which encodes the p16^INK4a^ cell cycle inhibitor^31^. Consistent with the earlier reports, we found a significant reduction in the percentage of TEC that incorporate BrdU in fetal and neonatal *Eed*^*CKO*^ thymi (Fig. 4a). Moreover, *Cdkn2a* expression was highly upregulated in *Eed*^*CKO*^ compared to control TEC (Fig. 4b). We also assessed apoptosis in the TEC compartment by quantifying the frequency of FOXN1+ cells that stained positively for cleaved caspase 3 (CC3) in control and *Eed*^*CKO*^ newborn thymi. Despite the overall low frequency of CC3+ cells due to rapid clearance of apoptotic cells, the data show an increased frequency of CC3+ TEC in *Eed*^*CKO*^ thymi (Fig. 4c).

Given that PRC2 activity is essential for proper differentiation of epithelial progenitors in lung, skin, heart and other organs^28,32,33^, we asked whether TEC differentiation was altered in *Eed*^*CKO*^ thymi. Immunohistochemical (IHC) analysis of keratin (K) staining patterns revealed defects in both cortical TEC (cTEC) and medullary TEC (mTEC) compartments. In the cortex, the K8+K5−K14− cTEC network was sparse in *Eed*^*CKO*^ compared to control newborn thymi (Fig. 4d). Mutant thymi also contained fewer and smaller medullary regions identified by the presence of K8−K5+K14+ mTEC. In addition, whereas there was a prominent population of K8+K5+ TEC surrounding the medullary regions in control thymi, this subset was relatively scarce in mutant thymi (Fig. 4d). Interestingly, it has been suggested that the K8+K5+ subset contains TEC with progenitor activity^34,35^. Defects in *Eed*^*CKO*^ TEC differentiation were also apparent in the data from flow cytometric analyses of TEC subsets. Consistent with the IHC results, there was a significant decrease in the percentage and number of *Eed*^*CKO*^ mTEC (CD45^−^EpCAM^+^UEA-1^+^Ly51^−^) (Fig. 4e,g,h). The number of mutant cTEC (CD45^−^EpCAM^+^UEA-1^-^ Ly51^+^), was marginally reduced, but did not achieve statistical significance, perhaps due to greater variability compared to mTEC counts (Fig. 4e,g,h). Additional evidence of defective differentiation was obtained from analysis of MHC class II (MHCII) expression, which is upregulated during maturation of postnatal cTEC and mTEC^36,37^. *Eed*^*CKO*^ thymi contain a notable expansion of TEC that express low levels of MHCII, a finding consistent with a block in TEC differentiation (Fig. 4f).

Collectively, IHC and flow cytometry analysis demonstrate that aberrant TEC proliferation, survival and differentiation contribute to the progressive loss of both cTEC and mTEC compartments in *Eed*^*CKO*^ thymi.

### A high frequency of DEG in *Eed*^*CKO*^ TEC are shared with DEG in *iTbx1* TEC

**Figure 5:**
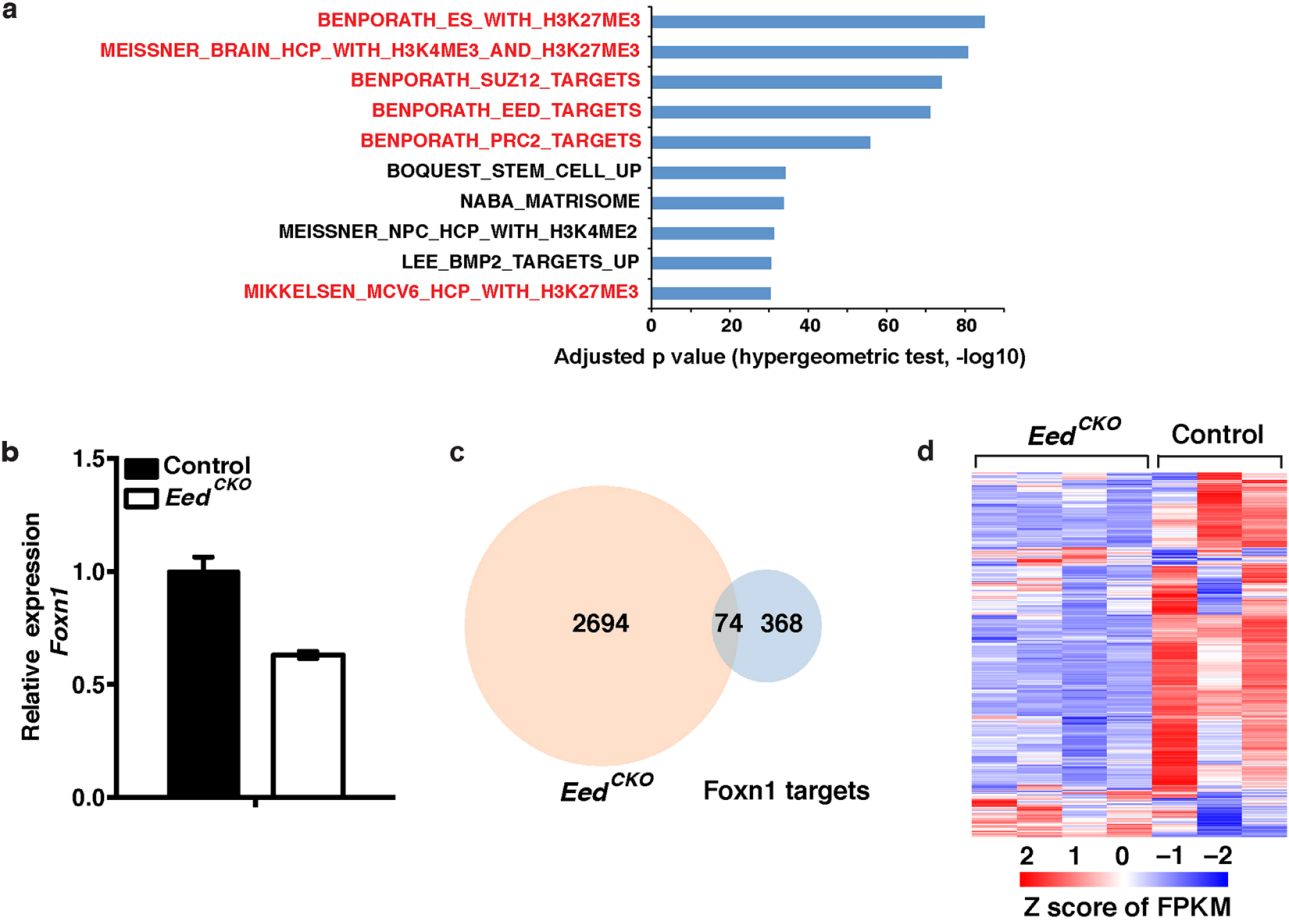
*Eed*^*CKO*^ DEG includes PRC2 and *Foxn1* target genes. (**a**) Bar graph showing DEG in *Eed*^*CKO*^ TEC are significantly enriched for PRC2, EED, SUZ12 and H3K27me3 gene targets (red). The top 10 significantly enriched gene sets are plotted; the length of bar represents −log10 of the adjusted p value calculated using a hypergeometric test^57^. (**b**) qRT-PCR analysis of *Foxn1* expression in E17.5 *Eed*^*CKO*^ TEC isolated by FACS sorting. Expression in *Eed*^*CKO*^ TEC is normalized to control TEC. Each sample was analyzed in triplicate and the experiment was repeated twice. (**c**) Venn diagram showing the number of overlapping genes between DEG and *Foxn1* target genes. (**d**) Heatmap of *Foxn1* target genes in *Eed*^*CKO*^ and control TEC expressed as Z score of FPKM.

Comparative gene expression profiling was performed on *Eed*^*CKO*^ and control TEC that had been isolated from fetal thymic lobes at E17.5, prior to the decline in TEC cellularity. The RNA-seq data revealed 2,768 DEG (FDR < 0.05 and FC > 2). As expected from the transcriptional silencing function of PRC2, the vast majority (82%) of *Eed*^*CKO*^ DEG were upregulated (Table S3). In addition, DEG from *Eed*^*CKO*^ mutants were significantly enriched for PRC2, EED, SUZ12 and H3K27me3 target genes (Fig. 5a).

In contrast to the overall pattern of increased gene expression, *Foxn1* was downregulated in *Eed*^*CKO*^ TEC (Fig. 5b and Table S3). This finding suggested that *Foxn1* is an indirect target of PRC2 activity. Consistent with decreased *Foxn1* expression, *Eed*^*CKO*^ mutant DEG contained 74 *Foxn1* target genes of which 71 were found to be downregulated (Fig. 5c and Table S4). When this analysis was expanded to include genes with FC of >1.5, we found that 206 of 442 high confidence *Foxn1* target genes^21^ that were expressed in *Eed*^*CKO*^ mutant TEC were downregulated (Fig. 5d). *Foxn1* target genes include niche factors that are required to support the entry and/or survival of CD4− CD8− CD25− CD44+ c-kit+ early thymocyte progenitors (ETP). RNA-seq data showed that transcripts for several *Foxn1* regulated ETP niche factors (DLL4, CXCL12, and CCL25) were reduced in *Eed*^*CKO*^ TEC (Fig. S3e). The deteriorating TEC compartment in *Eed*^*CKO*^ postnatal mice failed to support normal thymopoiesis as shown by the profound decrease in thymus cellularity (Fig. 2). Flow cytometric analysis revealed that the distribution of thymocyte subsets was not altered in mutant thymi despite the reduction in the number of thymocytes at all maturation stages (Fig. S3a,b). The marked decline in the number of ETP (Fig. S3c,d) suggests that failure of the mutant thymus to provide adequate ETP microenvironmental niches likely contributes to the decrease in thymocyte cellularity. Neither the number nor frequency of non-T lineage hematopoietic cells was increased in the mutant thymi.

**Figure 6:**
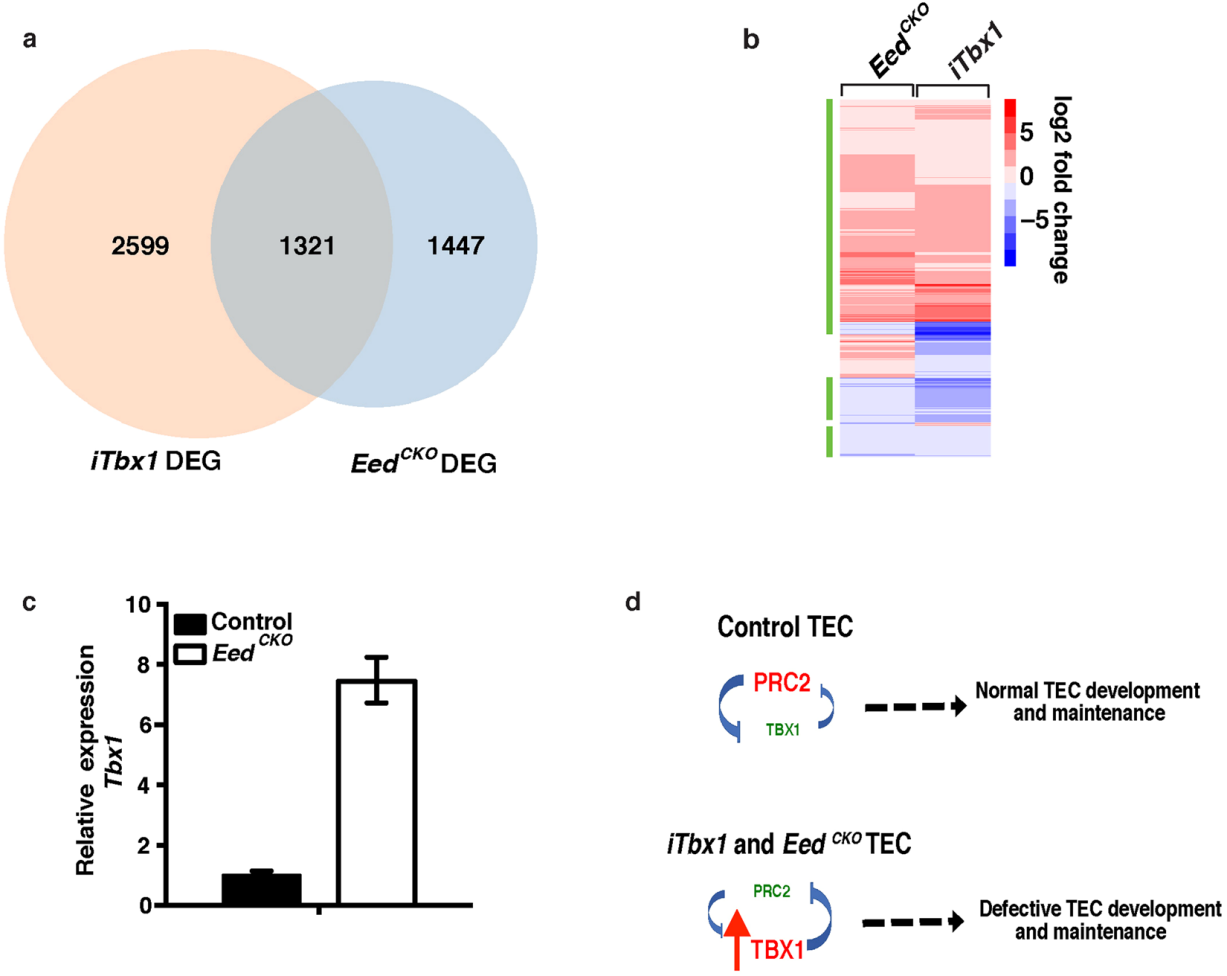
Overlap between *Eed*^*CKO*^ DEG and iTbx1 DEG. (**a**) Venn diagram showing overlap between *iTbx1* DEG and *Eed*^*CKO*^ DEG. (**b**) Heatmap of log2 fold change of 1321 overlapping DEGs in *iTbx1* and *Eed*^*CKO*^ TEC. The green bar denotes genes that are regulated in the same direction in *iTbx1* and *Eed*^*CKO*^ mutant TEC relative to their respective control TEC. (**c**) qRT-PCR analysis of *Tbx1* expression in E17.5 *Eed*^*CKO*^ TEC isolated by FACS sorting. Expression in *Eed*^*CKO*^ TEC is normalized to control TEC. Each sample was analyzed in triplicate and the experiment was repeated twice. (**d**) Working model showing reciprocal relationship between PRC2 activity and *Tbx1* expression.

Given that expression of PRC2 target genes is elevated in both *iTbx1* and *Eed*^*CKO*^ TEC and that both mutants have defects in TEC proliferation and differentiation, we compared the extent of overlap between the DEG from each mutant strain. Interestingly, 1,321(48%) of the *Eed*^*CKO*^ DEG are also differentially expressed in *iTbx1* mutants (Fig. 6a, Table S5). Moreover, 1148 (87%) of the common DEG were differentially regulated in the same direction, with the majority of shared genes being upregulated (Fig. 6b). Interestingly, *Tbx1* is one of the DEG that is upregulated in *Eed*^*CKO*^ TEC (log2 ratio *Eed*^*CKO*^/control FPKM = 2.19). The increase in *Tbx1* expression in *Eed*^*CKO*^ TEC was verified by qRT-PCR analysis of FACS sorted TEC (Fig. 6c). Increased transcription of *Tbx1* in *Eed*^*CKO*^ TEC implies the presence of a feedback loop between PRC2 and *Tbx1*. We propose a model in which PRC2 activity is one mechanism that restrains *Tbx1* expression in wildtype TEC (Fig. 6d). Therefore, loss of PRC2 activity in *Eed*^*CKO*^ mutant TEC would permit increased *Tbx1* expression. In the *iTbx1* mutants, *Tbx1* is expressed due to *Foxn1*^*Cre*^-mediated activation of the *R26*^*iTbx1*^ allele. In either case, decreased PRC2 activity would then enhance inappropriate gene expression leading to impaired TEC development and/or maintenance.

### *Eed* deletion activates expression of non-TEC transcriptional signatures

**Figure 7:**
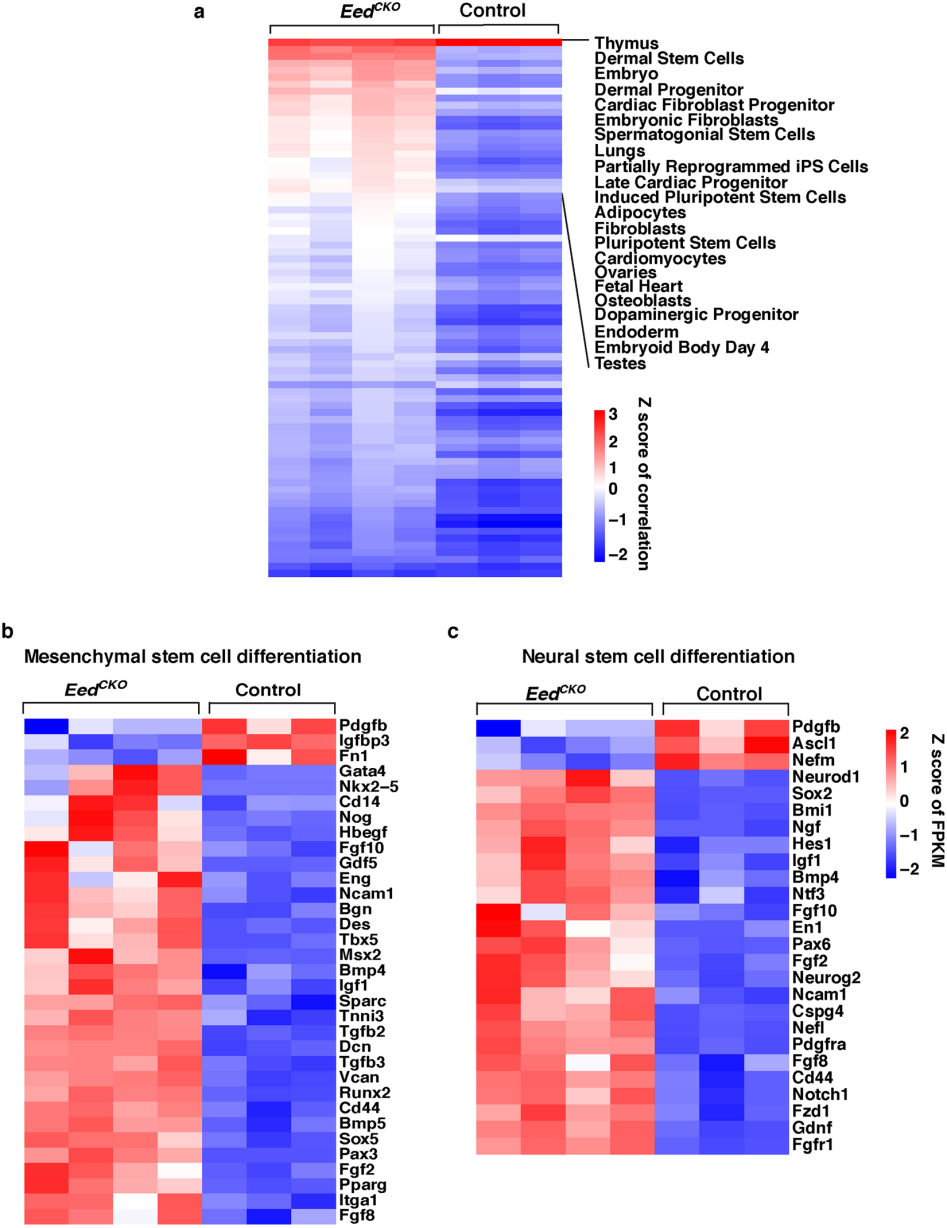
*Eed* deletion activates expression of non-TEC transcriptional signatures. (**a**) Gene expression correlation of tissue-specific genes in *Eed*^*CKO*^ and control TEC. AltAnalyze LineageProfiler was used to correlate the transcriptional profiles of lineage-specific markers with gene expression in *Eed*^*CKO*^ and control TEC. The log_2_FPKM values of *Eed*^*CKO*^ DEG were used as input to identify differences of lineage-specific expression profiles. Correlation coefficients were z-score normalized to plot the heatmap. (**b**) Mesenchymal stem cell differentiation pathway genes and (**c**) neural stem cell differentiation pathway genes were collected from PathCards (http://pathcards.genecards.org/). From these genes, *Eed*^*CKO*^ DEG were selected and z-score normalized log_2_FPKM values were used to plot the heatmaps.

A primary function of PRC2 is to maintain transcriptional repression of inappropriate lineage genes^38–40^. To determine if alternative lineage genes are upregulated in *Eed*^*CKO*^ TEC, we compared gene expression profiles of fetal *Eed*^*CKO*^ TEC with a curated database of lineage-specific genes from various tissues using AltAnalyze Lineage Profile (Fig. 7a). The transcriptomes of control and *Eed*^*CKO*^ TEC are most highly correlated with thymus, the tissue of origin. However, there was also a high degree of correlation between the transcriptional programs of *Eed*^*CKO*^ TEC and progenitor or stem cells from various lineages (Fig. 7a). For example, gene expression patterns typically associated with mesenchymal or neural stem cells are highly upregulated in *Eed*^*CKO*^ TEC (Fig. 7b,c). Sustained expression of non-TEC lineage progenitor genes could play a role in the increasingly severe phenotype observed in *Eed*^*CKO*^ postnatal thymi. Consistent with this notion, the vast majority of epithelial cells in the thymic remnants from young adult *Eed*^*CKO*^ mice co-expressed K5 and K14, but were K8 negative, a phenotype that is characteristic of immature basal cells in skin and other organs (Fig. S4). Furthermore, the K5+K14+ cells in adult *Eed*^*CKO*^ thymi did not express detectable levels of FOXN1. Taken together, these data indicate that deleting *Eed* in TEC progenitors abrogates transcriptional restraints that normally restrict expression of alternative lineage gene signatures leading to transformation of the highly compartmentalized thymic epithelial network into a primitive, poorly differentiated epithelial remnant.

## Discussion

Organ development and homeostasis depend on transcriptional and epigenetic networks that regulate the differentiation, outgrowth and maintenance of tissue specific progenitors. Precise control of gene expression patterns requires coordinated crosstalk between transcriptional and epigenetic factors. We previously reported that ectopic expression of *Tbx1* in TEC progenitors impairs TEC development and thymus organogenesis^7^. The present investigation shows that inappropriate expression of *Tbx1* in TEC progenitors alters epigenetic control of gene expression by interfering with PRC2-mediated transcriptional silencing. This finding suggests that downregulation of *Tbx1* in TEC progenitors is necessary to maintain optimal PRC2-mediated transcriptional repression. In support of this notion, we show that inactivating PRC2 activity by deleting *Eed* progressively impairs TEC proliferation, differentiation and lineage restriction. Thus, PRC2 plays a critical role in regulating TEC development and homeostasis.

TBX1 is a member of the T-box family of transcription factors that regulate gene expression directly via binding to DNA and indirectly via interactions with SMADs and other transcription factors or co-factors^41–43^. TBX1 has also been implicated in epigenetic control of gene expression based on interactions with Ash21 and Baf60a, which are protein subunits of Trithorax group and SWI/SNF chromatin remodeling complexes respectively^44,45^. In addition, a recent study demonstrated that TBX1 enhances H3K4me1 modification of target genes by interacting with and recruiting histone methyltransferases^46^. Moreover, inhibiting histone demethylase activity partially rescued the cardiac defects that develop in mutants expressing reduced *Tbx1* levels^46^. Collectively these reports demonstrate that TBX1 is a multifunctional protein that regulates gene expression by epigenetic as well as transcriptional mechanisms [reviewed in^47^]. Additional evidence for crosstalk between TBX1 and epigenetic regulation is provided in this investigation, which demonstrates that PRC2 target genes are upregulated in *iTbx1* TEC.

Transcriptional profiling revealed that approximately 50% of the DEG in *Eed*^*CKO*^ TEC overlap with DEG in *iTbx1* TEC and that >85% of the shared DEG are differentially regulated in the same direction. These results suggest that ectopic TBX1 impairs TEC development^7^, at least in part, by blocking PRC2-mediated gene suppression. In support of this premise, other groups have shown that PRC2 regulates progenitor cell fate specification, proliferation and differentiation in various developmental contexts^38–40^. The present investigation extends PRC2-mediated regulation of these processes to the TEC compartment by showing that *Eed* deletion leads to defective TEC proliferation, survival and differentiation accompanied by a progressive loss of TEC cellularity. An earlier report showed that deleting *Cbx4*, a PRC1 component, decreased the proliferation of fetal TEC^12^. However, to our knowledge, the current investigation is the first study to report a link between PRC2 and TEC proliferation.

Foxn1^Cre^-mediated deletion of *Eed* disrupts differentiation as well as outgrowth of TEC progenitors. The block in mTEC development as well as the increased frequency of immature MHCII^lo^ cTEC in the *Eed*^*CKO*^ TEC compartment support a stringent requirement for PRC2 function in TEC differentiation. Interestingly, *Foxn1* is one of the relatively few DEG that is downregulated as a consequence of *Eed* depletion. As FOXN1 functions in a dosage dependent manner to regulate cTEC and mTEC differentiation^4,48,49^, the decrease in *Foxn1* expression is likely a major contributing factor to aberrant differentiation of *Eed*^*CKO*^ TEC. However, given that *Foxn1* expression is reduced, but not abrogated, and that PRC2 regulates a wide range of target genes, it is likely that additional transcriptional alterations contribute to impaired differentiation of *Eed*^*CKO*^ TEC.

Deposition of repressive H3K27me3 epigenetic marks on chromatin is the canonical mechanism by which the PRC2 complex silences gene expression^10,22^. Consistent with this well-established function, the vast majority of DEG are upregulated and H3K27me3 levels are decreased in *Eed*^*CKO*^ TEC. However, recent studies have shown that PRC2 components can also exert non-canonical activity. For example, EZH2 regulates actin polymerization by methylating non-histone proteins such as Vav1 and Talin^50,51^. EZH2 also regulates development of the NKT lineage by directly methylating the transcription factor PLZF, leading to its ubiquitination and degradation^52^. EED can also function independently of its canonical role as a scaffolding protein that stabilizes the PRC2 complex. In cardiomyocytes, EED binds and stimulates the activity of histone deacetylases (HDACs)^53^. This non-canonical function of EED is particularly relevant to the present investigation because HDAC3 activity is required for the induction of a transcriptional program that promotes mTEC lineage development^54^. Therefore, a reduction in HDAC3 activity due to *Eed* deletion may contribute to the defective mTEC differentiation we observed in *Eed*^*CKO*^ mutants.

Deletion of *Eed* also upregulated expression of non-TEC genes, particularly those expressed in progenitor cells from other tissues. The appearance of transcriptional signatures characteristic of alternative lineages is not surprising given previous reports showing that the PRC2 complex transcriptionally silences expression of inappropriate lineage genes in a tissue specific manner^38–40^. It seems likely that the progressively aberrant thymic phenotype observed in *Eed*^*CKO*^ mice is associated with the observed derepression of alternative lineage genes. This process eventually converts the thymus into a non-functional epithelial remnant that fails to provide the appropriate microenvironmental signals needed for T cell production. Consistent with this notion, newborn *Eed*^*CKO*^ thymi contain severely reduced numbers of thymocyte subsets at all maturation stages including the earliest T cell progenitors, indicating a failure of the TEC compartment to recruit and/or support survival and differentiation of T cell progenitors. Therefore, PRC2 activity in the TEC compartment is indispensable for maintaining a functional TEC network that is capable of supporting robust thymopoiesis and T cell output.

A previous report showed that deleting *Ezh2* to inactivate PRC2 function in muscle satellite progenitors increased *Tbx1* expression^55^. Similarly, we demonstrate here that *Tbx1* expression is upregulated in TEC when PRC2 function is impaired as a consequence of deleting *Eed*. We also find that ectopic expression of *Tbx1* diminishes PRC2 activity in *iTbx1* TEC. These reciprocal results imply the presence of a negative feedback loop between *Tbx1* and PRC2. We suggest a model (Fig. 6d) in which PRC2 activity is necessary to transcriptionally silence *Tbx1* expression in TEC. In this model, the inappropriate expression of *Tbx1* interferes with PRC2-mediated transcriptional repression. The mechanisms responsible for the proposed regulatory loop are not yet clear. Whereas RNA-seq and qRT-PCR analyses of *Eed*^*CKO*^ TEC demonstrate that PRC2 suppresses *Tbx1* gene expression, RNA-seq analysis of *iTbx1* TEC did not reveal altered expression of genes that encode major PRC2 subunit components (data not shown). Therefore, we speculate that the presence of ectopic TBX1 in *iTbx1* TEC interferes with the activity of the PRC2 complex rather than suppressing expression of PRC2 component genes.

Collectively, the results of this study identify a functional link between *Tbx1* expression and PRC2 activity and suggest that interference with PRC2 function is one mechanism by which ectopic *Tbx1* expression dysregulates the development of TEC progenitors. Moreover, we show that *Eed* deletion adversely affects TEC proliferation, differentiation and lineage restriction demonstrating that the multifunctional PRC2 complex is indispensable for development and maintenance of a functional TEC microenvironment.

## Materials and Methods

### Mice

MD Anderson Cancer Center IACUC approved the research performed with mice. The approval number is 00001097-RN00. MD Anderson is accredited by the Association for Assessment and Accreditation of Laboratory Animal Care (AAALC). The AAALC number is 000183. All experimental procedures were carried out according to the protocols approved by Institutional Animal Care and Use Committee. After termination of the experiments, animals were euthanized in a CO_2_ chamber.

*Eed*^*fl/fl*^ ^25^, *R26*^*iTbx1/*+^ ^7^ and *Foxn1*^*Cre*^ mice^26^ were previously described. Each of these strains is maintained on a C57Bl6/J background in the Department of Epigenetics and Molecular Carcinogenesis at the MD Anderson Cancer Center Science Park in accordance with the guidelines set forth by the Association for the Accreditation of Laboratory Animal Care. Timed pregnancies were set up in the evening and monitored daily thereafter. The day of vaginal plug was considered embryonic day (E) 0.5. Morphological cues were used to determine embryonic stage. The sequences of primers used for genotyping tail DNA are listed in Table S6.

### Immunohistology and immunofluorescence staining

For immunohistology, embryos or thymi were fixed for 1 hr in 4% paraformaldehyde (PFA), washed in PBS, dehydrated in an ethanol gradient, cleared in xylene for 5 min and embedded in paraffin. Serial 10 μm sections were cut on a Leica ThermoShandon Finesse 325 microtome, washed, and stained with hematoxylin and eosin (H&E) according to standard techniques. For immunofluorescence, embryos or neonatal thymi were fixed in 4% PFA, dehydrated in sucrose and embedded in OCT. Serial 10 μm sections were air dried, washed and incubated overnight at 4 °C in 120 μl containing primary antibody, 10% donkey serum and 0.05% Tween-20. Slides were washed three times in PBS containing 0.05% Triton X-100 and incubated with 120 μl of secondary antibody in PBS containing 10% donkey serum and 0.05% Tween-20 for 1 hr at room temperature, in the dark. Slides were washed in PBS, incubated with 4′, 6-diamidino-2-phenylindole (DAPI) for 5 min at room temperature, and washed in PBS, mounted with ProLong® Gold antifade reagent (Life Technologies) and coverslipped. Primary antibodies used included goat anti-Foxn1 (Santa Cruz, G-20), rabbit anti-H3k27me3 (Active Motif), rabbit anti-H3k4me1 (Active Motif), goat anti-K14 (Santa Cruz), rabbit anti-K5 (Covance) rat anti-K8 (Troma-1 Developmental Studies Hybridoma Bank). Secondary antibodies were conjugated with either Alexafluor 488, Alexafluor 647 or CyTM3 (Jackson Immunoresearch). Slides were imaged on either a Leica DMI 6000B fluorescence microscope or a Zeiss LSM 880 laser-scanning confocal microscope.

To analyze cellular MFI in images, individual cell surfaces were segmented and masked based on Foxn1 fluorescence, and the MFIs of H3k27me3, H3k4me1, or Ezh2 signals were quantified using Imaris v9.1 (Bitplane). Control and mutant samples were processed in parallel and imaged at the same detector exposure so that the means and distributions of their MFIs can be directly compared.

### Flow cytometry

For TEC analysis fetal thymi were digested with 0.125% Collagenase (Sigma) and 0.1% DNase (Roche) as previously described^7^. For analysis of thymocyte subsets, E15.5 thymi were pressed through a 70 μm strainer (Fisher). Cells were stained with fluorochrome-conjugated antibodies in FACS buffer (PBS pH 7.2, 0.005 M EDTA, 2% FBS) for 15 minutes on ice and washed with FACS buffer. Propidium iodide (Invitrogen) was added (0.5 μg/ml) to each sample prior to analysis to exclude dead cells. Cells were stained with the following antibodies purchased from Biolgend or eBioscience: anti-CD326 (clone G8.8), anti-I-A/I-E (clone M5/114.15.2), biotinylated anti-Rat Ly51 (Clone 6C3), anti-CD25 (clone PC61), anti-CD117 (clone 2 B8), anti-CD44 (clone IM7), anti-CD8α (clone 53-6.7), anti-CD45 (clone 30-F11), anti-CD4 (clone RM4-5). To exclude erythrocytes, granulocytes, dendritic cells, macrophages and NK cells from the thymocyte analyses, we used the following antibodies, all conjugated to PE-Cy5 were purchased: TER-119, CD11c (cloneN418), CD11b (clone M1/70), NK-1.1 (clone PK136) and Ly-6G (clone RB6-8C5). Cells were analyzed or sorted on a FACS Aria II (BD Science). Data were analyzed using FlowJo software (Tree Star).

### Proliferation and apoptosis assays

BrdU (1 mg in 100 μl) was injected by the intraperitoneal (ip) route into pregnant females and E17.5 embryos were harvested after 90 minute chase. Alternatively, newborn pups were ip injected with BrdU (200 μg in 20 μl) and thymic lobes were harvested after 2 hrs. Thymi were digested with 0.5 WU/ml Liberase ^TM^ (Roche) containing 0.1% DNASE (Roche). Cells were stained with antibodies to surface proteins, followed by fixation and permeablization and staining with anti-BrdU (BD Pharmingen^TM^ BrdU Flow Kit). To determine the frequency of TEC apoptosis, 4% PFA fixed frozen thymus sections were stained with antibody to cleaved caspase 3 (Cell Signaling Technology) and FOXN1.

### Quantitative Reverse Transcriptase Polymerase Chain Reaction (qRT-PCR)

EpCAM+ CD45− TEC were sorted directly into Trizol (Ambion). RNA was isolated using chloroform and linear acrylamide (Ambion) and resuspended in nuclease free water prior to cDNA synthesis. First-strand cDNA was synthesized using a SuperScript® III First-Strand cDNA synthesis kit (Invitrogen). For qRT-PCR analysis of m*Foxn1*, *mEed*, m*Tbx1*, we used both TaqMan® Gene Expression Master Mix (Applied Biosystems) and SYBR green Master mix and used an ABI 7900HT Fast Real-Time PCR System. Primer sequences are listed in Table S1. Controls included samples with no RT or no template. All experiments included technical triplicate and were repeated at least twice. Data was analyzed using the ΔΔCt method. Expression levels in mutant TEC are shown relative to control TEC after normalization to *mHmbs and mHprt* housekeeping genes.

### RNA-seq analysis

Total RNA was obtained from *iTbx1* and control (E15.5) or *Eed*^*CKO*^ and control (E17.5) TEC that had been isolated by FACS sorting for EpCAM+ CD45− cells from thymus single cell suspensions prepared as described above. RNA quality was assessed using a NanoDrop and Bioanalyzer. RNA libraries were generated in the Next-Gen Sequencing Core in the Virginia Harris Cockrell Cancer Research Center at MD Anderson Science Park from 2–4 mutants and 3 controls of each strain using the Illumina TruSeq total RNA kit according to the manufacturer’s instructions. Each library was sequenced on an Illumina HiSeq2500.

### RNA-seq data mapping and analysis

Paired-end RNA-seq reads were aligned to Ensembl GRCm38 reference genome using TopHat v2.1.0 with parameters –library-type fr-firststrand. Accepted alignments were counted using the featureCounts with parameter -s 2. Then the DESeq2 R package was used for DEG. The Z-score normalized FPKM was used to plot heatmaps with R package pheatmap. DEG were based on a false discovery rate (FDR) of <0.05 and a fold change (FC) of >2. For GSEA, MSigDB v5.0 c2 gene sets were downloaded from http://software.broadinstitute.org/gsea/msigdb. The Piano R package was used to compute hypergeometric test on the enrichment of DEGs with MSigDB v5.0 c2 gene sets. Multiple testing was corrected by FDR (false discovery rate). GSEA with ENCODE histone modification database was performed with web-based tool Enrichr^56^. The list of iTbx1 DEG was used to test for enrichment and Benjamin-Hochberg method was used to correct for multiple testing according to default Enrichr setting.

For lineage-specific gene expression AltAnalyze LineageProfiler was used to infer the most likely cell types and tissues from RNA-seq data of *Eed*^*CKO*^ mutants and controls. Specifically, a database containing the most robust lineage specific expressed genes was constructed. Correlation scores were then derived by comparing the input gene expression profile with this lineage-specific database. Log_2_FPKM of *Eed*^*CKO*^ mutants and controls were used as input. Z scores of correlation efficient were used as output and heatmap plot. Known markers for mesenchymal cell differentiation and neural differentiation were used to perform supervised analysis of lineage-specific gene expression. Known markers were downloaded from http://pathcards.genecards.org/.

## Electronic supplementary material

Supplementary Information

Table S1

Table S2

Table S3

Table S4

Table S5

## Data Availability

All data generated or analyzed during this study are included in this published article and its Supplementary Information files.

## References

[1] Gordon J, Manley NR (2011). Mechanisms of thymus organogenesis and morphogenesis. Development.

[2] Boehm T (2008). Thymus development and function. Curr Opin Immunol.

[3] Abramson J, Anderson G (2017). Thymic Epithelial Cells. Annu Rev Immunol.

[4] Blackburn CC (1996). The nu gene acts cell-autonomously and is required for differentiation of thymic epithelial progenitors. Proc Natl Acad Sci USA.

[5] Liu Z, Yu S, Manley NR (2007). Gcm2 is required for the differentiation and survival of parathyroid precursor cells in the parathyroid/thymus primordia. Dev Biol.

[6] Zamisch M (2005). Ontogeny and regulation of IL-7-expressing thymic epithelial cells. J Immunol.

[7] Reeh KA (2014). Ectopic TBX1 suppresses thymic epithelial cell differentiation and proliferation during thymus organogenesis. Development.

[8] Manley NR, Selleri L, Brendolan A, Gordon J, Cleary ML (2004). Abnormalities of caudal pharyngeal pouch development in Pbx1 knockout mice mimic loss of Hox3 paralogs. Dev Biol.

[9] Vitelli F, Morishima M, Taddei I, Lindsay EA, Baldini A (2002). Tbx1 mutation causes multiple cardiovascular defects and disrupts neural crest and cranial nerve migratory pathways. Hum Mol Genet.

[10] Piunti A, Shilatifard A (2016). Epigenetic balance of gene expression by Polycomb and COMPASS families. Science.

[11] Chen T, Dent SY (2014). Chromatin modifiers and remodellers: regulators of cellular differentiation. Nature reviews. Genetics.

[12] Liu B (2013). Cbx4 regulates the proliferation of thymic epithelial cells and thymus function. Development.

[13] Candi E (2007). DeltaNp63 regulates thymic development through enhanced expression of FgfR2 and Jag2. Proc Natl Acad Sci USA.

[14] Senoo M, Pinto F, Crum CP, McKeon F (2007). p63 Is essential for the proliferative potential of stem cells in stratified epithelia. Cell.

[15] Mardaryev AN (2016). Cbx4 maintains the epithelial lineage identity and cell proliferation in the developing stratified epithelium. J Cell Biol.

[16] Sansom SN (2014). Population and single-cell genomics reveal the Aire dependency, relief from Polycomb silencing, and distribution of self-antigen expression in thymic epithelia. Genome Res.

[17] Greer EL, Shi Y (2012). Histone methylation: a dynamic mark in health, disease and inheritance. Nat Rev Genet.

[18] Margueron R (2009). Role of the polycomb protein EED in the propagation of repressive histone marks. Nature.

[19] Riising EM (2014). Gene silencing triggers polycomb repressive complex 2 recruitment to CpG islands genome wide. Mol Cell.

[20] Zhang Z (2005). Tbx1 expression in pharyngeal epithelia is necessary for pharyngeal arch artery development. Development.

[21] Zuklys S (2016). Foxn1 regulates key target genes essential for T cell development in postnatal thymic epithelial cells. Nat Immunol.

[22] Margueron R, Reinberg D (2011). The Polycomb complex PRC2 and its mark in life. Nature.

[23] Simon JA, Kingston RE (2013). Occupying chromatin: Polycomb mechanisms for getting to genomic targets, stopping transcriptional traffic, and staying put. Mol Cell.

[24] Montgomery ND (2005). The murine polycomb group protein Eed is required for global histone H3 lysine-27 methylation. Curr Biol.

[25] Xie H (2014). Polycomb repressive complex 2 regulates normal hematopoietic stem cell function in a developmental-stage-specific manner. Cell Stem Cell.

[26] Gordon J (2007). Specific expression of lacZ and cre recombinase in fetal thymic epithelial cells by multiplex gene targeting at the Foxn1 locus. BMC Dev Biol.

[27] Ezhkova E (2009). Ezh2 orchestrates gene expression for the stepwise differentiation of tissue-specific stem cells. Cell.

[28] He A (2012). Polycomb repressive complex 2 regulates normal development of the mouse heart. Circ Res.

[29] Jadhav U (2016). Acquired Tissue-Specific Promoter Bivalency Is a Basis for PRC2 Necessity in Adult Cells. Cell.

[30] Woodhouse S, Pugazhendhi D, Brien P, Pell JM (2013). Ezh2 maintains a key phase of muscle satellite cell expansion but does not regulate terminal differentiation. J Cell Sci.

[31] Sherr CJ, Roberts JM (1999). CDK inhibitors: positive and negative regulators of G1-phase progression. Genes Dev.

[32] Ezhkova E (2011). EZH1 and EZH2 cogovern histone H3K27 trimethylation and are essential for hair follicle homeostasis and wound repair. Genes Dev.

[33] Snitow ME (2015). Ezh2 represses the basal cell lineage during lung endoderm development. Development.

[34] Klug DB (1998). Interdependence of cortical thymic epithelial cell differentiation and T-lineage commitment. Proc Natl Acad Sci USA.

[35] Wong K (2014). Multilineage potential and self-renewal define an epithelial progenitor cell population in the adult thymus. Cell Rep.

[36] Gray D, Abramson J, Benoist C, Mathis D (2007). Proliferative arrest and rapid turnover of thymic epithelial cells expressing Aire. J Exp Med.

[37] Nowell CS (2011). Foxn1 regulates lineage progression in cortical and medullary thymic epithelial cells but is dispensable for medullary sublineage divergence. PLoS Genet.

[38] Adam RC, Fuchs E (2016). The Yin and Yang of Chromatin Dynamics In Stem Cell Fate Selection. Trends Genet.

[39] Aloia L, Di Stefano B, Di Croce L (2013). Polycomb complexes in stem cells and embryonic development. Development.

[40] Conway E, Healy E, Bracken AP (2015). PRC2 mediated H3K27 methylations in cellular identity and cancer. Curr Opin Cell Biol.

[41] Fulcoli FG, Huynh T, Scambler PJ, Baldini A (2009). Tbx1 regulates the BMP-Smad1 pathway in a transcription independent manner. PLoS One.

[42] Papangeli I, Scambler PJ (2013). Tbx1 genetically interacts with the transforming growth factor-beta/bone morphogenetic protein inhibitor Smad7 during great vessel remodeling. Circ Res.

[43] Papaioannou VE (2014). The T-box gene family: emerging roles in development, stem cells and cancer. Development.

[44] Chen Li, Fulcoli Filomena Gabriella, Ferrentino Rosa, Martucciello Stefania, Illingworth Elizabeth A., Baldini Antonio (2012). Transcriptional Control in Cardiac Progenitors: Tbx1 Interacts with the BAF Chromatin Remodeling Complex and Regulates Wnt5a. PLoS Genetics.

[45] Stoller JZ (2010). Ash2l interacts with Tbx1 and is required during early embryogenesis. Exp Biol Med (Maywood).

[46] Fulcoli FG (2016). Rebalancing gene haploinsufficiency *in vivo* by targeting chromatin. Nat Commun.

[47] Baldini A, Fulcoli FG, Illingworth E (2017). Tbx1: Transcriptional and Developmental Functions. Curr Top Dev Biol.

[48] Chen L, Xiao S, Manley NR (2009). Foxn1 is required to maintain the postnatal thymic microenvironment in a dosage-sensitive manner. Blood.

[49] Nehls M (1996). Two genetically separable steps in the differentiation of thymic epithelium. Science.

[50] Gunawan M (2015). The methyltransferase Ezh2 controls cell adhesion and migration through direct methylation of the extranuclear regulatory protein talin. Nat Immunol.

[51] Su IH (2005). Polycomb group protein ezh2 controls actin polymerization and cell signaling. Cell.

[52] Vasanthakumar A (2017). A non-canonical function of Ezh2 preserves immune homeostasis. EMBO Rep.

[53] Ai, S. *et al*. EED orchestration of heart maturation through interaction with HDACs is H3K27me3-independent. *Elife* **6**10.7554/eLife.24570 (2017). 10.7554/eLife.24570 PMC5400508 28394251

[54] Goldfarb Y (2016). HDAC3 Is a Master Regulator of mTEC Development. Cell Rep.

[55] Juan AH (2011). Polycomb EZH2 controls self-renewal and safeguards the transcriptional identity of skeletal muscle stem cells. Genes Dev.

[56] Chen EY (2013). Enrichr: interactive and collaborative HTML5 gene list enrichment analysis tool. BMC Bioinformatics.

[57] Subramanian A (2005). Gene set enrichment analysis: a knowledge-based approach for interpreting genome-wide expression profiles. Proc Natl Acad Sci USA.

