# Supplementary material for: Polycomb Repressive Complex 2 is essential for development and maintenance of a functional TEC compartment
Source: Sci Rep. 2018 Sep 25;8:14335. doi: 10.1038/s41598-018-32729-z (PMC6156232; doi:10.1038/s41598-018-32729-z)
Supplement: Supplementary file 1 — Supplementary Information [file 41598_2018_32729_MOESM1_ESM.pdf]

Supplementary Information for:

Polycomb Repressive Complex 2 is essential for development and maintenance of a functional TEC compartment

Nandini Singarapu[1], Keyue Ma[2], Kaitlin A.G. Reeh[1], Jianjun Shen[1], Jessica N. Lancaster[3], Song Yi[4] Huafeng Xie[5,6], Stuart Orkin[5,6,7,8], Nancy R. Manley[9], Lauren I.R. Ehrlich[3,10], Ning Jiang[2], Ellen R. Richie[1*]

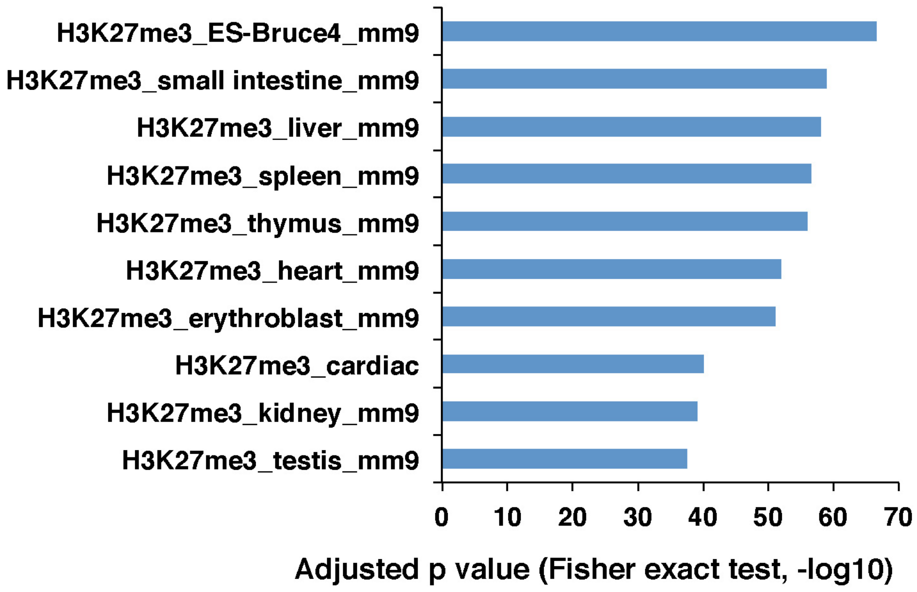
H3K27me3_ES-Bruce4_mm9
H3K27me3_small intestine_mm9
H3K27me3_liver_mm9
H3K27me3_spleen_mm9
H3K27me3_thymus_mm9
H3K27me3_heart_mm9
H3K27me3_erythroblast_mm9
H3K27me3_cardiac
H3K27me3_kidney_mm9
H3K27me3_testis_mm9
0
10
20
30
40
50
60
70
Adjusted p value (Fisher exact test, -log10)

Figure S1. *iTbx1* mutant DEG are enriched for genes containing H3K27me3 marks. The ENCODE Histone Modification database was used to assess *iTbx1* DEG for enrichment of genes containing H3K27me3 marks. The top 10 significantly enriched gene sets are plotted. The length of each bar is–log10 of the adjusted p value calculated using the Fisher exact test and Benjamin-Hochberg corrected for multiple testing.

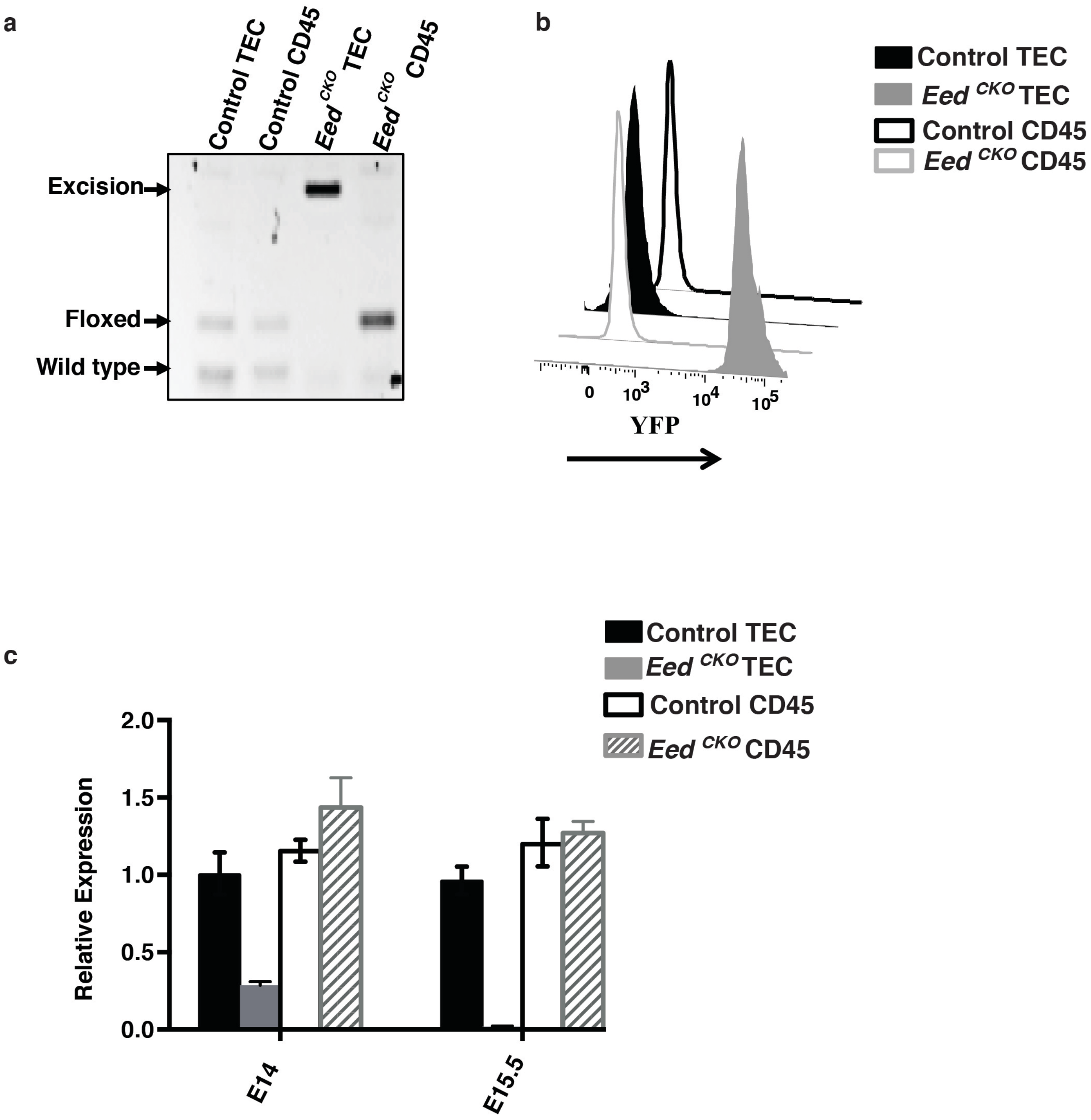
a
Control TEC
Control CD45
Eed CKO TEC
Eed CKO CD45
Excision
Floxed
Wild type
b
Control TEC
Eed CKO TEC
Control CD45
Eed CKO CD45
0
10^3
10^4
10^5
YFP
c
Control TEC
Eed CKO TEC
Control CD45
Eed CKO CD45
Relative Expression
2.0
1.5
1.0
0.5
0.0
E14
E15.5

Figure S2. Deletion of *Eed* in *Foxn1*$^{Cre/+}$;*Eed*$^{fl/fl}$ TEC. (a) PCR analysis of *Eed* deletion in FACS sorted thymocytes (CD45+ EpCAM- cells) and TEC (CD45- EpCAM+ cells). (b) Flow cytometric analysis of YFP expression by control and *Eed*^{CKO} TEC and thymocytes (c) qRT-PCR analysis of *Eed* expressed by E14.5 and E15.5 TEC and thymocytes that were isolated by FACS sorting.

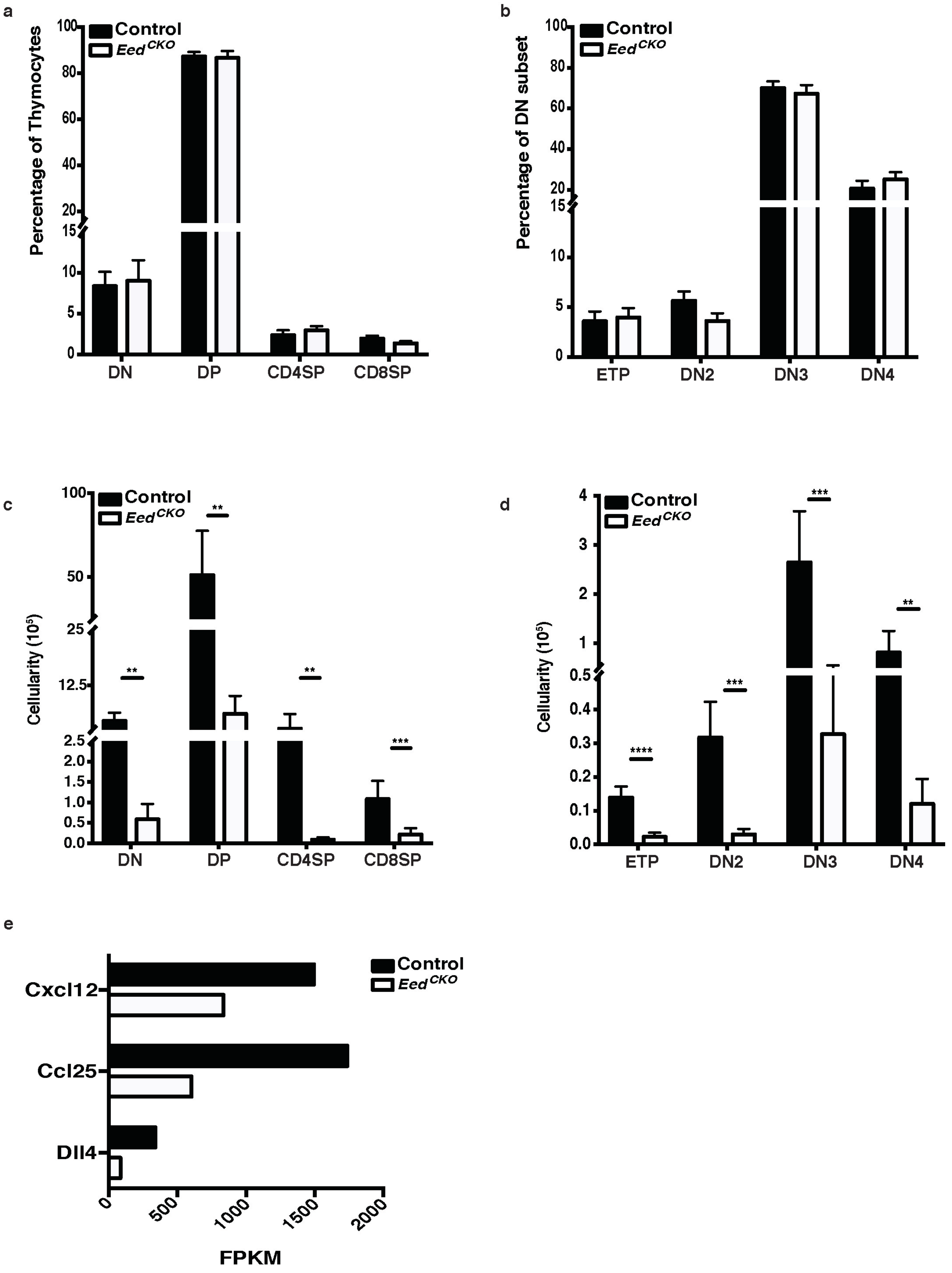
a
Control
Eed^CKO
Percentage of Thymocytes
100
80
60
40
20
15
10
5
0
DN
DP
CD4SP
CD8SP
b
Control
Eed^CKO
Percentage of DN subset
100
80
60
40
20
15
10
5
0
ETP
DN2
DN3
DN4
c
Control
Eed^CKO
Cellularity (10^5)
100
50
25
12.5
2.5
2.0
1.5
1.0
0.5
0.0
**
**
**
***
DN
DP
CD4SP
CD8SP
d
Control
Eed^CKO
Cellularity (10^5)
4
3
2
1
0.5
0.4
0.3
0.2
0.1
0.0
****
***
***
**
ETP
DN2
DN3
DN4
e
Control
Eed^CKO
Cxcl12
Ccl25
Dll4
0
500
1000
1500
2000
FPKM

Figure S3. The number, but not frequency, of thymocyte subsets is adversely affected by deleting *Eed* in the TEC compartment. (a) Percentage and (c) number of DN (CD4-CD8-), DP (CD4+CD8+), CD4SP (CD4+CD8-) and CD8SP (CD4-CD8+) thymocyte subsets in P0 control and Eed^{CKO} thymi. (b) Percentage and (d) number of ETP (c-kit+ CD25-), DN2 (c-kit+ CD25+), DN3 (c-kit- CD25+) and DN4 (c-kit- CD25-) subsets in P0 control and Eed^{CKO} thymi. Electronic gates were set to exclude non-T lineage and TCR positive DN cells. (e) RNA-seq data showing expression of genes encoding ETP niche factors Cxcl12, CCl25, and Dll4 in control and Eed^{CKO} TEC. Results are shown as fragments per kilobase of transcript per million mapped reads (FPKM).

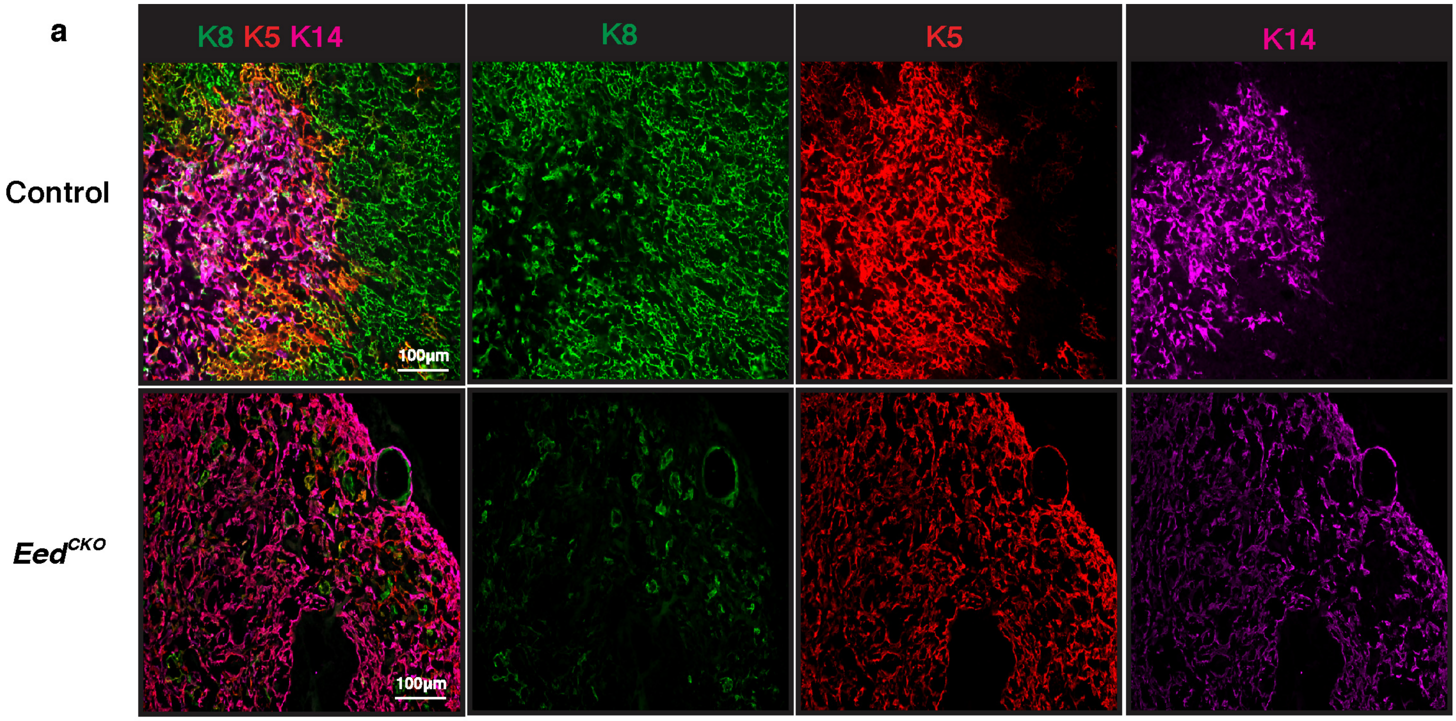
a
K8 K5 K14
K8
K5
K14
Control
EedCKO
100µm
100µm

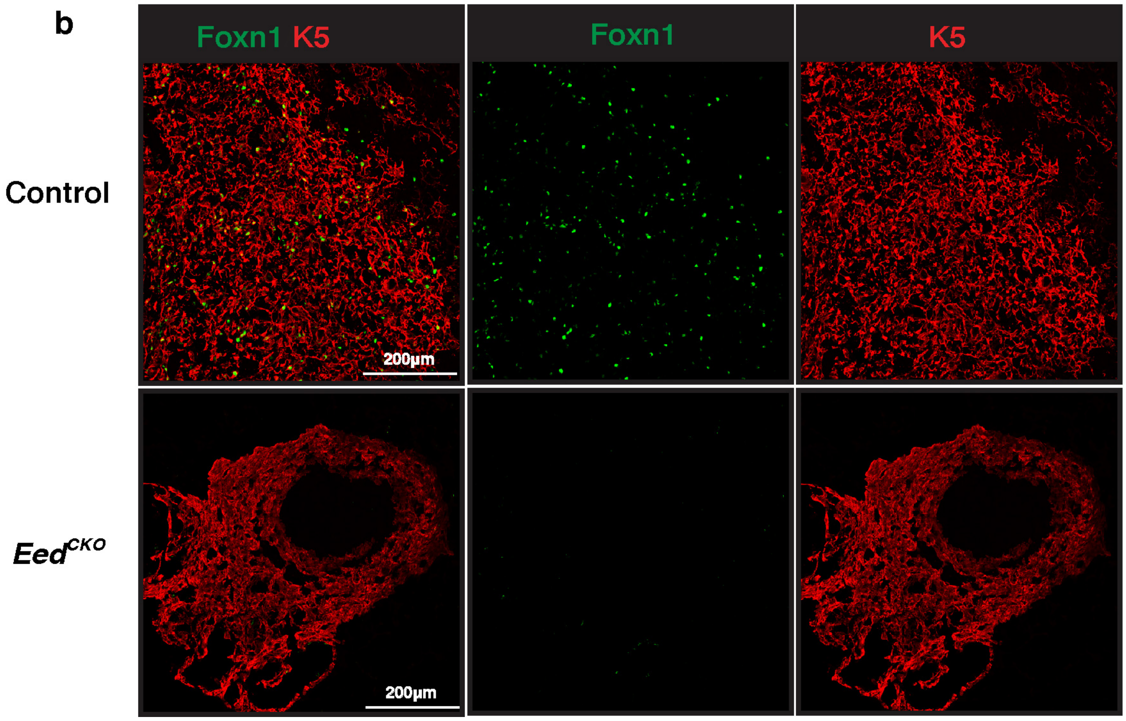
b
Foxn1 K5
Foxn1
K5
Control
EedCKO
200µm
200µm

Figure S4. Primitive basal-like phenotype of epithelial cells remaining in adult *Eed*^{CKO} thymic remnants.

Frozen sections from 4 week old control or *Eed*^{CKO} thymi were stained for (a) K8 (green), K5 (red) and K14 (blue) or (b) K5 (red) and FOXN1 (green). White bar indicates scale (100 μm).

Table S6. Primers used in this investigation

Primers	Sense sequence	Anti-sense sequence
**Genotyping Primers**		
Eed-WT	CTACGGGCAGGAGGAAGAG	GGGGGAGAGGGAGTTGTC
Eed-Floxed	CTACGGGCAGGAGGAAGAG	CCACATAGGCTCATAGAATTG
Cre	AGGTTCGTTCACTCATGGA	TCGACCAGTTTAGTTACCC
pRosa26-WT	AAAGTCGCTCTGAGTTGTTAT	GGAGCGGGAGAAATGGATATG
pRosa26-Tbx1	AAAGTCGCTCTGAGTTGTTAT	GCGAAGAGTTTGTCCTCAACC
**qRT-PCR Primers**		
Foxn1	TGACGGAGCACTTCCCTTAC	GACAGGTTATGGCGAACAGAA
mEed	CAACTGTGGGAAGCAACAGA	ATAGAGGGTGGCTGGTGTTG
mHprt	CCTCCTCAGACCGCTTTTT	AACCTGGTTCATCATCGCTAA
mHmbs	TCCCTGAAGGATGTGCCTAC	ACAAGGGTTTTCCCGTTTG
